# Enteritis as initial manifestation of systemic lupus erythematosus in early pregnancy: A case report

**DOI:** 10.1097/MD.0000000000010401

**Published:** 2018-04-27

**Authors:** Anna Maria Bellou, Dominik Bös, Guido Kukuk, Ulrich Gembruch, Waltraut Maria Merz

**Affiliations:** a Department of Obstetrics and Prenatal Medicine; b Department of Internal Medicine; c Department of Radiology, University Hospital Bonn, Bonn, Germany.

**Keywords:** lupus enteritis, pregnancy, systemic lupus erythematosus

## Abstract

**Rational:**

Lupus enteritis is a rare, severe complication of systemic lupus erythematosus (SLE). We report of a patient who presented with enteritis as manifestation of new-onset SLE during the first trimester of pregnancy.

**Patients concerns:**

The 23-year nulliparous patient was admitted to a district hospital with abdominal pain, nausea, vomiting and bloody diarrhea at a gestational age (GA) of 10 weeks. Her symptoms improved with symptomatic treatment and she was discharged a few days later. At 15 weeks’ of gestation she was readmitted. Her lab results revealed mild anemia and thrombocytopenia. Ascites, renal failure and proteinuria developed. An infectious cause was suspected, but stool samples and urine cultures were negative. Diagnostic work-up included abdominal ultrasound, gastro- and sigmoidoscopy, magnetic resonance imaging (MRI), and diagnostic laparoscopy. Ultrasound and MRI revealed dilated, fluid-filled small bowel loops, and increased colonic wall diameters. Mucosal edema and petechiae were detected by sigmoidoscopy, and histopathologic examination of the biopsies revealed erosive inflammation. Due to progressive deterioration she was transferred to our center. In addition to ascites, pleural and pericardial effusions had developed.

**Diagnosis:**

Diagnosis of SLE was finally established at GA 16 after an autoimmune workup revealed positive antinuclear, anti- Sm, anti-dsDNA and anti-U1RNP antibodies. An interdisciplinary team was set up for her management. She was commenced on corticosteroids; response was only partial and necessitated addition of cyclosporine. The further clinical course was complicated by anemia, chest wall shingles, hypertension, and progressive cervical shortening. Serial ultrasound and Doppler examinations revealed notching of the uterine arteries with raised pulsatility indices and fetal growth restriction.

**Intervention:**

At GA 35 abdominal pain reoccurred; a decision for delivery was taken. An apparently healthy fetus was delivered by cesarian section with good Apgar scores and pH (2100g, 9. percentile). The postoperative / postnatal course was unremarkable.

**Outcomes:**

New-onset SLE during pregnancy is rare, as is lupus enteritis. To our knowledge, our case is the first report of a combination of both.

**Lessons:**

Diagnostic delay occurred a result of symptom overlap and limitations in diagnostic imaging. Interdisciplinary teamwork resulted in successful outcome for both, mother and fetus.

## 1 Introduction

Systemic lupus erythematosus (SLE) is a polymorphic disease, affecting preferentially women in their reproductive years.^[1]^ Maternal sequelae of SLE in pregnancy are predominantly determined by organ involvement and disease activity before the onset of pregnancy, and may encompass a favorable outcome for both, mother and fetus. Multiorgan involvement or lupus nephritis herald maternal and fetal complications, including renal function deterioration, preeclampsia, placental insufficiency, and preterm birth.^[2,3]^ Additionally, fetal prognosis is determined by type and level of maternal auto-antibodies, with the risk of irreversible destruction of the fetal conduction system and intrauterine demise.^[4]^ Flares may occur during pregnancy in up to 68% and require multidisciplinary teamwork for successful maternal and fetal-neonatal outcome.^[5]^

Initial SLE manifestation during pregnancy is exceedingly rare and may be misdiagnosed as preeclampsia or any other disease, depending on the organ manifestations.^[6]^ Autoimmune gastrointestinal complications do occur in patients with known SLE. A retrospective single-center analysis reports an incidence of 0.6%,^[7]^ and a recent review gives a mortality rate of 2.7% in lupus enteritis, predominantly from complications of perforation.^[8]^ Thus, lupus enteritis comprises a rare and serious complication of SLE, requiring swift diagnosis and prompt therapy. Clinical symptoms consist of abdominal pain, vomiting, diarrhea, and fever and may not only overlap with other causes of enteritis, but also with symptoms of early pregnancy. We report of a patient who presented with enteritis as manifestation of new-onset SLE during the first trimester of pregnancy. The case report involves patient consent and informed consent was given.

## 2 Case report

**Figure 1:**
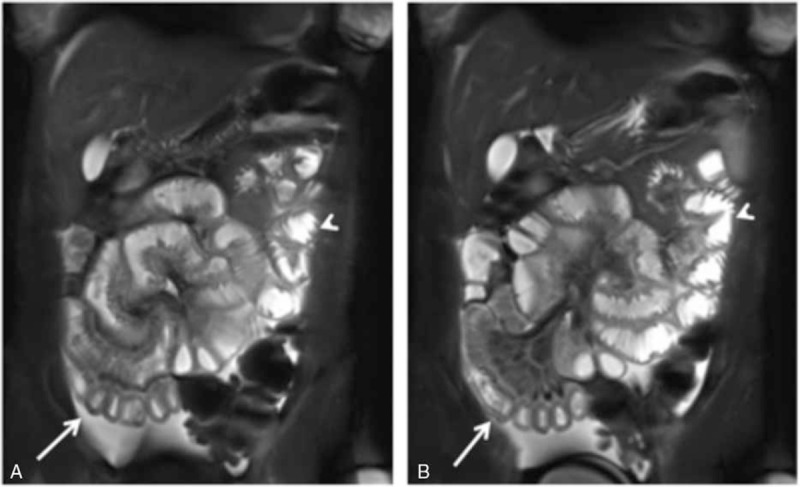
A, B: Abdomen MRI revealing dilated, fluid-filled small bowel loops and increased colonic wall diameters (arrows). MRI = magnetic resonance imaging.

The 23-year-old nulliparous patient was admitted to a district hospital with abdominal pain, nausea, vomiting, and bloody diarrhea at a gestational age of 10 weeks. Her symptoms improved with non-specific treatment and she was discharged a few days later. At 15 weeks of gestation she was readmitted with severe abdominal pain. Lab results revealed mild anemia and thrombocytopenia (see Table 1). Ascites and acute renal failure developed within a few days. An infectious cause was suspected, but stool samples and urine cultures were negative. Diagnostic work-up included abdominal ultrasound, upper gastrointestinal endoscopy, rectosigmoidoscopy, abdominal magnetic resonance imaging (MRI), and diagnostic laparoscopy. Ultrasound and MRI revealed dilated, fluid-filled small bowel loops, and increased colonic wall diameters (see Fig. 1A, B). Mucosal edema and petechiae were detected by rectosigmoidoscopy, and histopathologic examination of the biopsies described erosive inflammation.

**Table 1:**
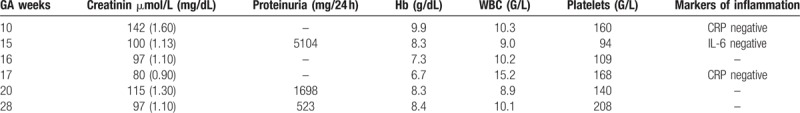
Hematologic and kidney function tests.

Due to progressive deterioration, the patient was transferred to our center. Apart from diffuse abdominal pain on palpation the physical examination was normal. She was hypertensive (180/90 mmHg), otherwise her vital signs were unremarkable. Ultrasound examination showed ascites, pleural, and pericardial effusions. The kidneys were of normal shape with increased echogenicity and lack of corticomedullary differentiation. Analysis of the urine sediment showed erythrocyturia and cellular casts, no white blood cells, and no evidence of urinary tract infection. Biochemical analyses during the course of pregnancy are listed in Table 1. The autoimmunology work-up on admission revealed positive antinuclear, anti-dsDNA, anti-Sm, and anti-U1RNP antibodies (see Table 2). Thus, diagnosis of SLE was finally established at 16 weeks of gestation.

**Table 2:**
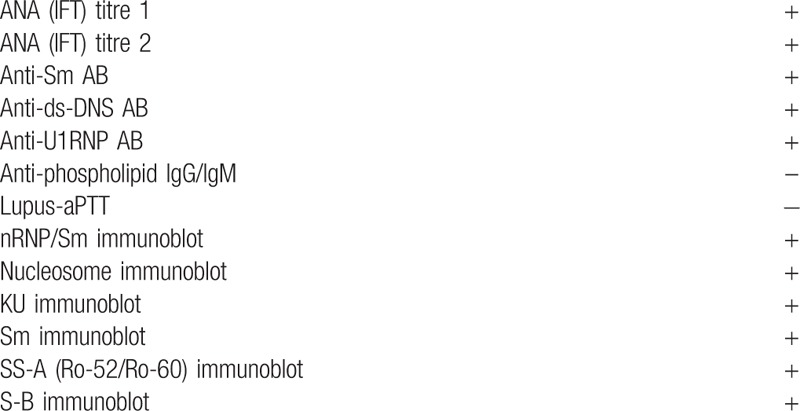
Autoimmunology work-up.

An interdisciplinary team was set up for her management. Her initial medication included furosemide, low-molecular-weight heparin, methyldopa, and omeprazole. In addition, steroid pulse therapy was initiated (methylprednisolone 250 mg/d intravenously for 5 days, followed by 100 mg/d orally). However, response was only partial; therefore, immunosuppressive therapy was intensified and ciclosporine was added (target through-level of 150 ng/mL). The intensified therapy resulted in a gradual recovery. Subsequently, hematologic and kidney function tests improved (see Table 1). Severe anemia necessitated transfusion of packed red cells (3 units). Steroid medication was tapered; her maintenance medication consisted of prednisolone (10 mg bd) and ciclosporine (125 mg bd). After stabilization, she was treated as outpatient. Further admissions were required during the course of pregnancy for chest wall shingles at 20 weeks of gestation and progressive cervical shortening at 26 weeks of gestation. Antihypertensive medication was suspended at 25 weeks of gestation, after normalization of her blood pressure.

**Figure 2:**
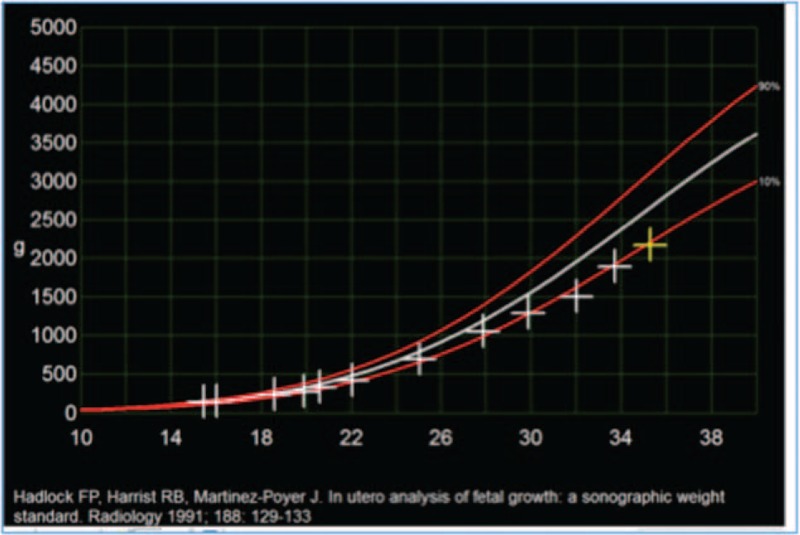
Serial biometry revealing development of fetal growth restriction.

Serial ultrasound and Doppler examinations revealed notching of the uterine arteries with raised pulsatility indices and fetal growth restriction (see Fig. 2)^[9]^. At 35 weeks of gestation abdominal pain reoccurred; a decision for delivery was taken. An apparently healthy male fetus with a birth weight of 2100 g (9^th^ percentile) was delivered by cesarean section with Apgar scores of 7/9/10 at 1, 5, and 10 minutes, respectively, and umbilical arterial pH of 7.31. The postoperative course was unremarkable, and both, mother and newborn, were discharged on postoperative day 6.

The patient came for follow-up in 2-weekly intervals for the first 3 months, and monthly thereafter. Renal function remained stable (serum creatinine around 89 μmol/L) without significant proteinuria. Though anti-dsDNA antibody levels initially decreased they failed to normalize, as did complement C3 and C4 levels. Kidney biopsy was finally performed 24 weeks after delivery. Histopathological examination showed focal segmental lupus nephritis as well as membranous lupus nephritis, that is, class III and V lupus nephritis.^[10]^ During the further course of disease development of hirsutism required a switch in the immunosuppressive regime. Mycophenolate mofetil (2 g/d) was started and well tolerated. Anti-dsDNA antibodies further decreased and complement C3 and C4-levels normalized.

## 3 Discussion

To our knowledge, our case is the first report of enteritis as initial manifestation of SLE, occurring in early pregnancy. The frequency of initial SLE manifestation in pregnancy is extremely low.^[6]^ Only case reports and small series are available which reveal a high maternal morbidity and—depending on the gestational age at disease onset—a dismal fetal outcome. Clowse and Grotegut,^[2]^ for example, found a diagnosis of SLE in 0.8% of deliveries in the USA between 2000 and 2003, with a maternal mortality of 1.1%. Compared with new-onset SLE in non-pregnant women the manifestation is more severe, and involvement of the renal and hematological system more common.^[11]^ The frequency of lupus enteritis is currently unknown; this may be due to the fact that definition and nomenclature are not concise, as is the underlying pathophysiologic mechanism; inflammatory and vascular causes are discussed.^[12]^

Diagnosis should be established after detailed assessment of clinical, laboratory, imaging, and histopathologic investigations. Diagnostic imaging consists of ultrasound and computed tomography, where characteristic features have been described. These include small bowel edema and “target sign,” the latter being abnormal bowel-wall thickening and enhancement. Mesenteric vessel engorgement with an increased number of visible vessels (“comb's sign”) is also mentioned as specific feature of lupus enteritis.^[7,8,13,14]^ Due to the presence of early pregnancy, MRI without contrast media was performed in our patient; it revealed unspecific inflammatory small bowel alterations. The occurrence of mesenteric microthrombi could not be verified.

Regarding treatment, lupus enteritis is usually steroid-responsive.^[8]^ In our patient ciclosporin had to be added for clinical stabilization, with concomitant recovery of kidney function, improvement of hematologic parameters, and blood pressure normalization. Particularly the latter is remarkable, given that hypertension usually worsens during the course of pregnancy.

## 4 Conclusion

In conclusion, our case highlights the diagnostic dilemma and consecutive delay in diagnosis establishment and treatment initiation as a consequence of 2 exceedingly rare, simultaneously occurring events. Interdisciplinary teamwork resulted in successful outcome for both, mother and fetus.

## Author contributions

**Conceptualization:** Anna Maria Bellou, Waltraut Maria Merz.

**Data curation:** Anna Maria Bellou, Waltraut Maria Merz.

**Investigation:** Anna Maria Bellou, Dominik Bös, Guido Kukuk, Waltraut Maria Merz.

**Project administration:** Anna Maria Bellou.

**Resources:** Anna Maria Bellou.

**Supervision:** Ulrich Gembruch.

**Writing—original draft:** Anna Maria Bellou.

**Writing—review and editing:** Waltraut Maria Merz.
