# Enteritis as initial manifestation of systemic lupus erythematosus in early pregnancy: A case report: Erratum

**DOI:** 10.1097/MD.0000000000010797

**Published:** 2018-05-25

In the article, “Enteritis as initial manifestation of systemic lupus erythematosus in early pregnancy: A case report”,^[1]^ which appears in Volume 97, Issue 17 of *Medicine*, Dr. Ulrich Gembruch's degree should appear as Prof. Dr. Med. and Dr. Guido Kukuk's degree should appear as PD. Dr. Med.
